# Evidence of elevated situational awareness for active duty soldiers during navigation of a virtual environment

**DOI:** 10.1371/journal.pone.0298867

**Published:** 2024-05-10

**Authors:** Leah R. Enders, Stephen M. Gordon, Heather Roy, Thomas Rohaly, Bianca Dalangin, Angela Jeter, Jessica Villarreal, Gary L. Boykin, Jonathan Touryan

**Affiliations:** 1 Human in Complex Systems Division, DEVCOM Army Research Laboratory, Aberdeen Proving Ground, Maryland, United States of America; 2 DCS Corporation, Alexandria, Virginia, United States of America; University of Leeds, UNITED KINGDOM

## Abstract

U.S. service members maintain constant situational awareness (SA) due to training and experience operating in dynamic and complex environments. Work examining how military experience impacts SA during visual search of a complex naturalistic environment, is limited. Here, we compare Active Duty service members and Civilians’ physiological behavior during a navigational visual search task in an open-world virtual environment (VE) while cognitive load was manipulated. We measured eye-tracking and electroencephalogram (EEG) outcomes from Active Duty (N = 21) and Civilians (N = 15) while they navigated a desktop VE at a self-regulated pace. Participants searched and counted targets (N = 15) presented among distractors, while cognitive load was manipulated with an auditory Math Task. Results showed Active Duty participants reported significantly greater/closer to the correct number of targets compared to Civilians. Overall, Active Duty participants scanned the VE with faster peak saccade velocities and greater average saccade magnitudes compared to Civilians. Convolutional Neural Network (CNN) response (EEG P-300) was significantly weighted more to initial fixations for the Active Duty group, showing reduced attentional resources on object refixations compared to Civilians. There were no group differences in fixation outcomes or overall CNN response when comparing targets versus distractor objects. When cognitive load was manipulated, only Civilians significantly decreased their average dwell time on each object and the Active Duty group had significantly fewer numbers of correct answers on the Math Task. Overall, the Active Duty group explored the VE with increased scanning speed and distance and reduced cognitive re-processing on objects, employing a different, perhaps expert, visual search strategy indicative of increased SA. The Active Duty group maintained SA in the main visual search task and did not appear to shift focus to the secondary Math Task. Future work could compare how a stress inducing environment impacts these groups’ physiological or cognitive markers and performance for these groups.

## Introduction

U.S. service members complete missions in dynamic and complex environments for sustained periods of time and must rapidly adapt to changes in tasking and mission priorities. For mission success, a keen sense of situational awareness (SA) is an invaluable skill, especially in dynamic modern combat scenarios. Effective SA involves the timely perception of key elements in the environment, comprehension of their meaning, and the ability to project implications for the near future [1, 2]. In the military, efficient visual scanning resulting in the rapid perception of increased threat/target and scenery/information change detection, increased threat neutralizations, and decreased friendly fire, are indicative of effective SA maintenance [3, 4]. This is especially critical in today’s battlefield as it is no longer linear in nature, but has been restructured with urbanized and dispersed fighting tactics [5]. Additionally, many current combat scenarios involve engaging with adversaries who are mingled covertly with Civilian noncombatants, severely impacting enemy engagement and increasing the need to maintain a high level of SA to rapidly discern and identify threats [5]. Given this evolution in warfare tactics, it is no surprise that the Department of the Army (DA) lists SA as a foundational skill and maintaining a high level of SA as integral in the reduction of human errors and combat causalities [6].

The DA has been working to integrate SA into military training over the last few decades to improve mission success and reduce errors in the field [5, 7–9]. As noted by Endsley [10], the ability to maintain a high level of SA in a task is dependent on skills, training, and experience, as well as previous understanding of the task details and objectives and the ongoing workload conditions. Fatigue, loud noises, and increased stress level negatively impact SA [11–13]. Therefore, training in capabilities to mitigate these effects (e.g., stress management) are thought to generate overall improvement in SA and task efficiency/success. Due to training that targets these elements of SA, military service members may gain expertise in these areas and exhibit a different pattern of SA compared to their Civilian counterparts. Differences in performance may thus be observed when completing SA-relevant tasks, such as visual searches.

The use of traditional questionnaire-based SA measures can be limiting, depending on the research paradigm. Studies with real-time SA measurement, such as the SA Global Assessment Technique (SAGAT) [2], would require pausing and interrupting the study, which could interfere with any simultaneous auditory tasks [14]. If administered post-data collection, such as the SA Rating Technique (SART) [15], the assessment depends heavily on recall. Real-time physiological measurement approaches provide an alternative to self-reporting SA questions and offer continuous and objective SA assessment. Also, physiological measurement offers an objective and task-relevant measurement of SA and provides critical information through underlying mechanisms (e.g., neural correlates). Eye tracking and electroencephalography (EEG) are two indirect physiological methods that give an objective assessment of SA [14, 16, 17]. Furthermore, these signals are easily integrated into virtual environments (VE), which allows the observation of more naturalistic behaviors and responses.

Eye tracking explicitly addresses Endsley’s [1] perception component of SA, providing direct means to measure, quantify, and assess an individual’s ability to perceive elements in their environment. For eye tracking, increased SA has been associated with an increase in the number of fixations and an increase in dwell time [17–20], as well as a decrease in fixation rate (fewer long fixations) and saccade magnitude [21] on the area of interest. Additionally, increased pupil size is indicative of increased cognitive load [22, 23] and hypervigilance during a task [24]. Also, increased peak saccade velocities are indicative of elevated levels of arousal [25]. Given this evidence, eye tracking provides an objective and task-relevant assessment of SA that is linked to underlying physiological mechanisms, such as neural processes, and can provide powerful insights into an individual’s level of SA and state (e.g., cognitive load).

Physiological measurement via EEG can also assess the cognitive processes associated with SA [26]. When considering EEG measures, changes in observed pre-task absolute alpha power significantly modulate neural mechanisms associated with SA task performance [27]. Additionally, the loss of SA is associated with activation of visual brain regions (e.g., primary visual cortex), as well as frontal, cingulate, and parietal brain regions that are associated with cognition during uncertainty [28]. Of specific interest in visual search studies investigating SA with EEG is the P300. The P300 is an event-related potential that occurs after approximately 300 ms (250–500 ms) following the onset of a low-probability or task-relevant stimulus [29]. The P300 response is particularly of interest in studies looking at visual processing and decision making [30, 31] where higher P300 amplitudes can be indicative of target acquisition [32], increased confidence in decision making, increased awareness, and increased engagement/effort during a task [30]. Given that SA involves the perception of elements in an environment [1, 2], eye tracking and EEG are powerful tools for capturing an objective, task-relevant measure of SA, without requiring in-experimentation interruptions. Additionally, both eye tracking and EEG provide critical links to the underlying mechanisms that are involved in maintaining SA (e.g., [18]; [27]). When paired with advances in machine learning and modeling, physiological data allows us to infer additional information about SA from data acquired in more naturalist and real-world environments that was previously considered too noisy [33].

We expect that Active Duty military service members will perform visual search tasks differently due to specific field training in visual search that is emphasized for service members (e.g., threat detection, target recognition, and so on). Previous work with even short visual search training paradigms have demonstrated differences in visual search outcomes for both military and Civilian populations (Table 1). For instance, using a short visual training simulation in a military and Civilian college student population, Haider and Frensch [34] found that training enabled an individual to distinguish between task-relevant and task-redundant information during a visual search task. Guznov et al. [35] demonstrated that when Civilian university students were trained in a target recognition, visual scanning, and cueing task during an unmanned aerial vehicle simulation, target search performance improved, target hits increased, and there was a reduction in false alarms when compared to no training. In another study, following a short training on identifying threats in baggage scanning in a virtual reality (VR) scenario, Civilians improved performance on the search task and improved performance was accompanied by an adaptation of visual scanning behaviors and P300 activation [36].

**Table 1:** Previous literature and measurements of situational awareness.

Author(s)	Impacts	Measurement	Metric	Results indicating changes in SA
**Haider and Frensch (1999)** [34]	Training Effects	Task Performance	Response Time	Response time in a visual search task was reduced post-training leading to faster capabilities of distinguishing between task-relevant and task-redundant information
**Winslow et al. (2013)** [36]	Training Effects	Task Performance	Response Time Accuracy	Individuals exhibited faster response times, higher hit rates, decreased misses, and increased false alarms post-training of a VR baggage scanning task
		Eye Behavior	Fixations	Successful identification of threats/distractors accompanied by reduced mean fixation durations, suggesting improvements in accuracy and efficiency
		EEG	P300	Higher P300 amplitudes post-training increased preparation/execution of a manual response to threat/distractor
**Guznov et al. (2017)** [35]	Training Effects	Task Performance	Accuracy	Training resulted in more target hits and fewer false alarms during a visual search task for targets in an unmanned aerial vehicle simulation
**Prytz et al. (2018)** [38]	Novice vs. Expert	Eye Behavior	Fixations	Experienced first responders allocated more attentional resources (longer dwell time but similar in number of fixations) to task-relevant information compared to novices
**Godwin et al. (2015)** [43]	Novice vs. Expert	Eye Behavior	Fixations	Individuals with military field experience displayed fewer eye fixations on threats prior to making movement decisions on a patrol task detecting explosive devices in static photographs, compared to novices
**Castner et al. (2022)** [39]	Novice vs. Expert	Eye Behavior	Saccades	Dental experts scanned environments with increased saccade lengths and saccade velocities compared to novices on a dental x-ray visual exploration task.
**Lanini-Maggi et al. (2021)** [42]	Novice vs. Expert	EEG	Engagement Index	Experts elicited a higher EEG-engagement index, suggesting increased focused attention in a task where controllers had to quickly identify an aircraft in a simulation, compared to novices
**Koh et al. (2011)** [48]	Novice vs. Expert	Eye Behavior	Fixations	Percentage of dwell times among expert nurses had a better fit with a model measuring optimal visual scanning strategies and less attentional shifts (interruptions) during final counts compared to novice nurses on a simulated surgery task
**Robinski and Stein (2013)** [41]	Novice vs. Expert	Eye Behavior	Fixations	Percent of fixations on targets increase with increased task demands for novice student pilots on a flight simulator task, but opposite found for expert pilots, suggesting novices may overlook areas of interest especially during high workload
**Nozima et al. (2017)** [44]	Training	Eye Behavior	Scan Path	When provided scan paths of expert military pilots, novice pilots significantly changed scan paths to mimic experts’ scanning patterns

Measurements used by selected prior work to assess properties of situational awareness (SA) between inexperienced and experienced individuals.

Given this work with trainings short in duration, it is not surprising to observe differences in visual behavior when comparing someone who is considered to have expertise (longer-term training and/or experience) in a skill area compared to a novice. A meta-analysis performed by Gegenfurtner, Lehtinen, and Sȁljȍ [37] used 296 different effect sizes to investigate how expertise impacts visual search. Findings from this study demonstrated that compared to novices, experts exhibit shorter individual fixation durations and more fixations on task-relevant areas (and reduced fixations on redundant areas), as well as longer saccades. Moreover, experts were quicker to fixate on task-relevant information, indicating they may process visual information faster and are seemingly more efficient with visual attention resources. When comparing experienced emergency service providers to novice witnesses in a visual search task involving accident and control imagery, Prytz, Norén, and Jonson [38] found that experienced first responders allocated more attentional resources (longer dwell time but a similar number of fixations) to task-relevant information compared to novices. In terms of specific visual scanning behaviors, experts have been known to scan environments with increased saccade lengths and saccade velocities compared to novices [39], which suggests that experts may rely more on parafoveal and peripheral visual field processing [40, 41]. In terms of cortical activation, Lanini-Maggi et al. [42] evaluated the impact of expertise on neural responses in a task that required controllers to quickly identify an aircraft in a simulated aircraft setting. They found that experts elicited a higher EEG-engagement index compared to novices, suggesting experts completed the task with increased focused attention.

Previous work specifically looking at how military expertise impacts visual search is sparse. One study found that military field experience was associated with increased performance, faster decision making, and reduced eye fixations on threats prior to decision-making compared to those with no field experience on a threat detection task with 2-D photographs [43]. Additionally, novice military pilots improved their visual scanning strategies by watching experts’ eye movements superimposed over a video of them solving a complex emergency flight procedure in simulation [44]. These results suggest not only that expertise itself can improve SA, but that novices may benefit and learn directly from the expertise of others to improve this skill.

Multitasking can have negative impacts on SA and a decrement in performance in multitasking is often observed due to limitations in attentional resources [45]. In military applications, multitasking while maintaining SA is a common requirement and any performance sacrifices could have exceptionally dire consequences when performing in-the-field tasks. For instance, as a Soldier’s workload increases, lethality capabilities decrease and can detrimentally impact survivability of that individual [46, 47] and potentially the survivability of fellow team members. Training in multitasking could reduce the negative impact of multitasking on SA and increase efficiency in task prioritization and improve performance under high task loads [34]. Thus, such SA training endured by military personnel could impact how they attentionally compensate during multitasking.

Experience has been shown to impact visual scanning and performance compensatory behaviors during manipulated workload. Differences in visual scanning techniques with increased workload have been noted between novices and experienced pilots where experienced pilots appeared to have a superior capability in processing the peripheral visual field, particularly in high workload conditions [41]. Experienced scrub nurses displayed certain visual attention management strategies (less task switching) and were superior in task performance compared to novice scrub nurses while multitasking during a final count of gauzes post-caesarian procedures [48]. Given military personnel’s expertise in maintaining a high level of SA under multitasking/increased workloads, it is expected that the military may adapt differently when workload is manipulated, and tracking changes in neurophysiological data could capture differences in cognitive processing compared to Civilian counterparts.

SA compensatory strategies during manipulated (e.g. multitasking, increase) workload are observed through changes in neurological and physiological outcomes including eye gaze behavior. Changes with manipulated workload in vision behavior outcomes appear highly task dependent, while others seem consistent regardless of the task. Generally, individuals appear to increase individual fixation durations [49–51] and increase pupil dilation [49–52] with increased workload. Blink and saccadic changes during increased workload are mixed and appear to be very task dependent, particularly with respect to workload increases in terms of increased demand of visual processing versus auditory processing [49, 50, 52, 53]. For instance, decreases in blink rate have accompanied increased demands on visual processing [49, 51, 54] but blink rate has been less reflective of changes in auditory processing [53]. In terms of neural activity, EEG measures of mean response latency negatively correlates with increased workload [55], as well as reduced P300 amplitude [53], demonstrating a reduction in SA as workload increases. Although heavily task dependent, both gaze behavior and EEGs are reflective of workload changes.

Research is sparse in comparing military and Civilian visual search patterns and neurological activation, particularly during an open-world navigation task. Many research paradigms investigating SA during visual searches used traditional visual search paradigms with static, 2D imagery. However, a real-life visual search (i.e., naturalistic eye movement in search of a target during self-paced and self-directed navigation) is generally conducted in more complex environments under much less controlled conditions. As technology advances, there has been an increase in the utilization of VEs to investigate human behavior during visual search tasks [31, 56–63]. A virtual desktop environment allows researchers to simulate dynamic scenarios that would otherwise not be possible (e.g., combat zones), simulate single and team scenarios, and measure physiological responses (e.g., eye behavior, neural activity, and heart rate) simultaneously. While visual search in VR or AR scenarios more closely replicate “real-life” by allowing physical ambulatory motion and greater free range of head movement, this technology currently still draws limitations for the simultaneous collection of physiological signals (e.g. EEG). Regardless of this limitation, using desktop VEs and physiological responses broadens our understanding of how humans disperse their visual attention and cognitive resources and can be applied to future work utilizing AR/VR technology. This information allows us to understand visual search patterns that may be unique to a certain population, such as military service members, and understand how such individuals may allocate their resources differently (than Civilians or other specialized fields), especially when cognitive load is manipulated. Ultimately, this information can be used for future integration of assistive technology in a combat team or to understand teaming dynamics and optimize team performance. This information can also be applied to future AR and VR experimental designs, particularly for populations (e.g. military, medical staff, police, transportation safety officers) where SA maintenance is a high priority and who undergo SA-targeted training.

Here, we examine differences in physiological responses (eye-tracking behavior and neurological activity) between an Active Duty military population and a Civilian population while actively navigating (self-directed) an open world desktop VE and completing a visual search task while cognitive load conditions are manipulated. We hypothesize that the Active Duty group will exhibit a unique visual behavior pattern (i.e., fixation and saccade differences) when scanning the environment for a certain target. Additionally, we investigate how the Active Duty group mitigates attentional resources when an additional cognitive load, an auditory Math Task, is added. We hypothesize that the Active Duty group will allocate attentional resources differently compared to the Civilian group when cognitive load is manipulated. The overall aim of this investigation is to capture a better understanding of how Active Duty service members, compared to Civilian counterparts, maintain SA and perform during a visual search task while navigating an open-world and understand how this changes when cognitive load is manipulated. We successfully demonstrate group differences in visual search strategy and the allocation of attentional resources, indicating that military training and experience impacts maintenance of SA.

## Materials and methods

### Participants

This study had 36 participants, including 21 active military participants in the Active Duty group (14 males and 2 females with a combined mean (M) age ± standard deviation (SD) = 36.7 ± 12.9 years) and 15 non-Active Duty military participants in the Civilian group (9 males and 6 females, with a combined M age ± SD = 35.3 ± 9.5 years). The Active Duty group participants were recruited from the Joint Base San Antonio (JBSA)—Fort Sam Houston Army base and the Civilian group participants were recruited as a part of a larger study from the Los Angeles (LA) location. Recruitment for this study occurred between the 11^th^ of November, 2019 and the 13^th^ of July, 2021. All participants in both groups went through the same experimental procedure. Due to prior findings from the larger data set for the Civilian group that found an effect of target condition on gaze behavior [58], the subset selected for the analyses presented here includes participants from both the Active Duty and Civilian groups that were assigned to search for the same target (i.e., Humvee). Participants in the Civilian group were compensated for their time. All participants were at least 18 years old, had normal hearing and corrected-to-normal vision and color vision, and were fluent in English. All participants signed an Institutional Review Board approved consent form, which was also reviewed and approved by the U.S. Army Combat Capabilities Development Command (DEVCOM) Army Research Laboratory (ARL) Human Research Protections Program [IRB#: ARL-19-122].

### Procedure

Participants completed four separate tasks: a Baseline Task, an old/new recognition task, a training VE, and the Primary Visual Search Task in the VE described in the following sections. Additionally, an online survey that included demographic information and questions about individual differences and states was administered throughout the experiment. Only the methods and results from the Primary Visual Search Task are included in this analysis.

#### Virtual environment and Primary Visual Search Task

Prior to navigating the testing VE, all participants practiced in a training VE where they were able to practice controlling their movement and the camera view (from a first-person perspective). The training VE was similar to the main VE in scenery but included different visual objects. To actively move through both the training and main VEs, a keyboard was used to navigate physically through the desktop environment. Participants used the “W”, “A”, “S”, and “D” keys to move forward, left, right, and backwards, respectively. They controlled the camera view with a computer mouse. Participants used these controls in the training VE to navigate a path and find and land on a bulls-eye target.

**Fig 1:**
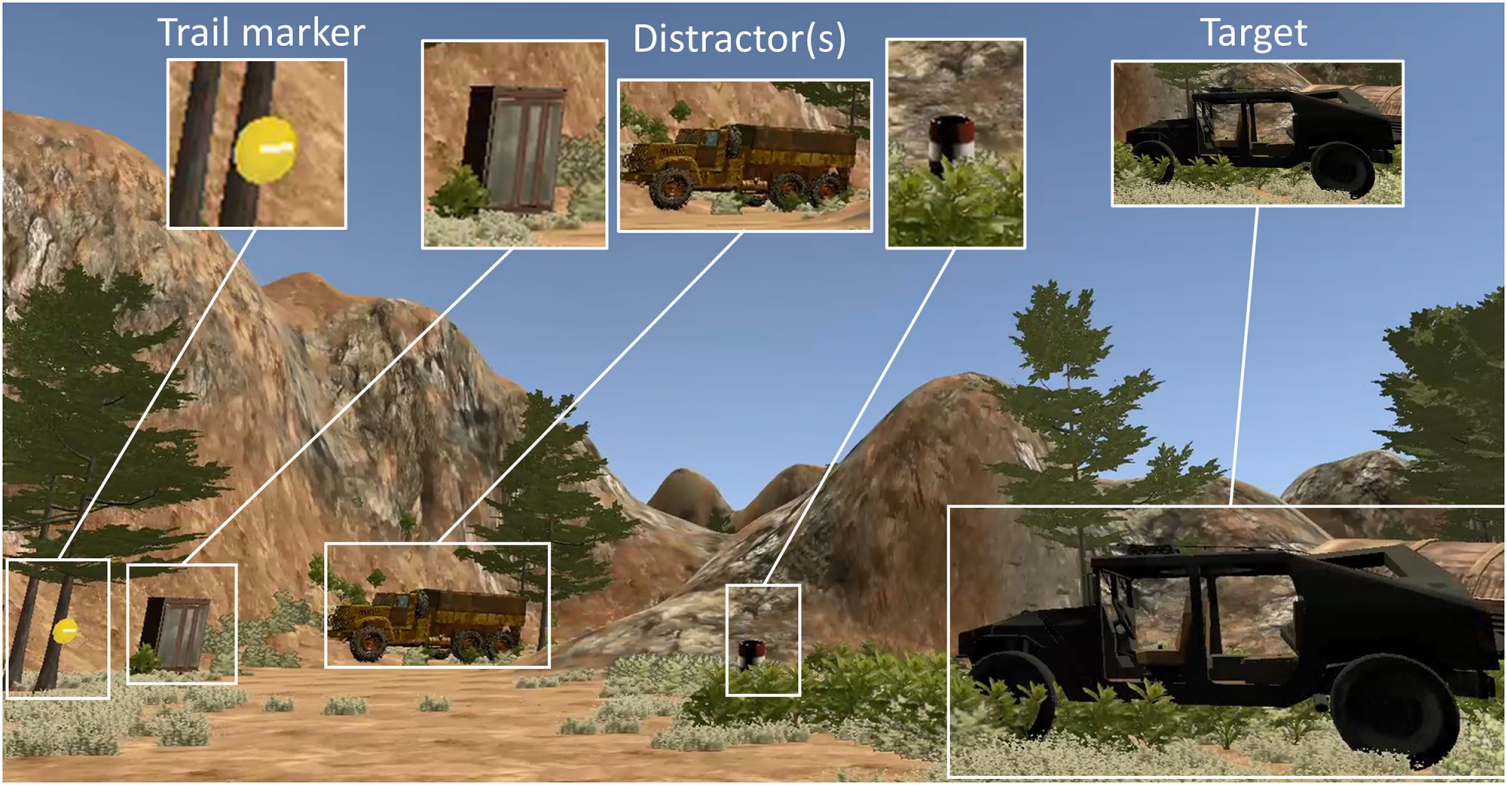
VE design and elements. A still image showing the VE and one of the Humvee targets that individuals in both groups searched for while navigating the environment. Trail markers (yellow circles on trees) were placed along the path to assist in navigation and distractors were placed throughout the environment along the path.

In the Primary Visual Search Task, participants searched a naturalistic canyon-like VE while searching and mentally counting the number of targets seen, Humvees (N = 15 total) (Fig 1). Navigation through the environment was self-paced and participants followed a general path, guided by trail markers (N = 19 total). Participants were instructed to move freely along the path observing all objects as they searched for the target object, a Humvee, which was placed in semi-regular intervals throughout the pathway. The same model was used for every Humvee, although the orientation of the Humvee along the path and distance with respect to the midline of the path was varied. In addition, sometimes the Humvee was partially occluded by terrain (i.e., brush, or trees) or distractors. Distractors were considered any object in the VE that was not the target (approximately N = 166), and included objects such as aircrafts, furniture, motorcycles, and barrels (to name a few) placed randomly in the environment. An in-depth description of the VE is discussed in greater detail elsewhere [58]. The maximum time allowed to navigate through the environment was 20 minutes. After finishing navigation of the VE, participants reported how many targets they counted during navigation.

#### Secondary Math Task

While navigating the VE and searching for targets, participants completed an additional auditory Math Task. This Math Task started approximately 8 minutes into navigation of the environment with the intention to increase cognitive load. An auditory voice prompt provided a set of three random numbers (e.g., 7, 20, 12). Participants were asked to mentally sum the numbers (e.g., 39) and provide a verbal response to the researcher when prompted. A total of three sets of numbers were presented in total, each separated by a period of 8 to 30 seconds. This secondary Math Task was designed based on other work investigating performance under divided attentional demand conditions [46–47]. The entire Math Task took approximately 90 to 120 seconds to complete and participants were encouraged to simultaneously continue searching for targets in the VE when listening, calculating, and answering the Math Task.

#### Testing setup

**Fig 2:**
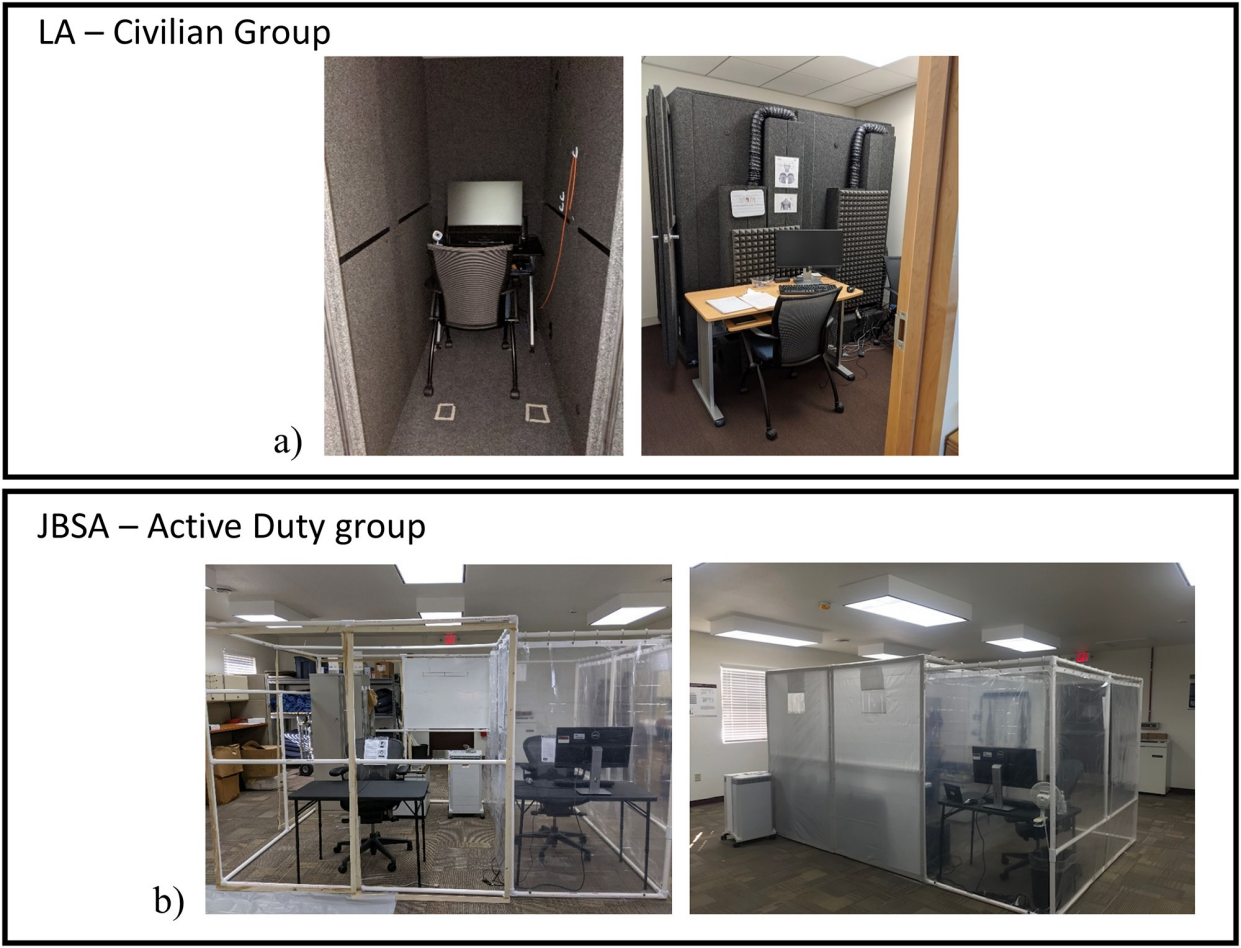
Experimental setup. The setup for data collection for the Civilian group in the Los Angeles (LA) area location (a) and for the Active Duty group at the Joint-Base San Antonio (JBSA) location (b).

Data collection with the Civilian group at the LA location was collected in a lab space where participants completed tasks in a Whisper room—an enclosed space intended to block out sound outside the chamber (Fig 2a, left). Experimenters sat outside the Whisper room with a mirrored desktop monitor to administer instructions and observe participants’ progress throughout the experiment (Fig 2a, right). Data collection with the Active Duty population at JBSA took place in a specially constructed experimental testing station designed for mitigating the risk of COVID-19, where participants completed their tasks in a space partitioned by a heavy plastic wall material (Fig 2b, left desk partition). Similar to the layout of the LA data collection, experimenters were outside the experimenter space with a mirrored desktop monitor (Fig 2b, right desk partition). Due to the COVID pandemic, the experimental setup at JBSA was intentionally designed to comply with COVID policies. Therefore, the setup included portable filter systems and customizable partitioned spaces, which stayed consistently placed throughout data collection.

Equipment at both locations involved the same components. Physiological data included eye tracking, EEG, electrooculography (EOG), and electrocardiography (EKG), although only the eye-tracking findings and a brief examination of the EEG results are discussed in this report. Eye tracking was collected using a Tobii Pro Spectrum (300 Hz) and the VE was built in Unity 3D environment (Unity Technologies) run on the standard Tobii Pro Spectrum monitor (EIZO FlexScan EV2451). Eye tracking was calibrated using a 5-point calibration protocol. Head movement was not restricted but participants were asked to maintain an upright sitting posture and to maintain proper alignment with the eye tracker. From the Tobii Pro Spectrum, gaze position and pupil size outcomes were measured. In addition, the Tobii Pro Spectrum provided the 3D gaze vector that was used to determine which object(s) in the VE that the participant was viewing while navigating the environment [58]. EEG, EOG, and EKG were measured using the 64-channel BioSemi ActiveView system.

#### Data processing of the gaze data in the Primary Visual Search Task

Gaze position, blinks, and saccades were detected and labeled for the Primary Visual Search Task according to the methods discussed in great detail in our previous work [58]. The following is a short description of how each of these variables were calculated for this analysis. Saccades were labeled as such if they had a duration of greater than 12 ms and a minimum inter-saccadic interval of 50 ms [64–66]. Gaps, missing samples in the gaze data that were between 50 and 500 ms in duration, were considered blinks. Fixations were only included if they were greater than 100 ms in duration [67–69]. For each fixation, the fixated object in the VE was defined as the object that had the highest percentage of collisions of the 3D gaze vector. We then removed fixations for which this maximum value was less than 10% (i.e., less than 10% of the samples from a single fixation were on the same object). Given that fixations represent stable points in the gaze field there was rarely more than 2 objects (plus terrain) intersected by the samples from a single fixation. Fixations exclusively on terrain or sky (i.e., no intersections with an object) were included in some analyses. For example, for the following distribution of gaze samples Object 1 (4%), Object 2 (5%), and Sky/Terrain (91%), the “fixated object” would be named the Sky/Terrain. If the distribution of gaze samples were changed to Object 1 (10%), Object 2 (25%), and Sky/Terrain (65%), the “fixated object” would be named as Object 2. This also enabled us to remove any distant objects that may have received a gaze vector collision, but were most likely too far away or too occluded in the VE to actually be perceived by the participant. For full justification of gaze-object methodology see [58].

#### Calculation of study variables with gaze data

To determine how well Military Status impacted participants’ performance on the Primary Visual Search Task, participants were asked to verbally report the number of targets they recalled seeing in the VE upon finishing navigation—referred to as the Self-Reported Target Count. To determine group differences in what objects were viewed in the VE, fixations on distractors, targets, and trail markers in the VE were extracted from the eye-tracker data and referred to as the Gaze-Validated Distractors, Gaze-Validated Targets, and Gaze-Validated Trail Markers. To further examine visual attention on objects in the VE, the Mean Number of Fixations, Mean Dwell Time, and Mean Distance on targets and on distractors were extracted from the eye-tracker data. The Mean Number of Fixations is defined as the total number of fixations that intersected with that object (target or distractor) in the VE. The Mean Dwell Time is the total time (sum of the duration of all individual fixations) on an object. The Mean Distance is the approximated “distance” (meters in the VE are approximate and do not reflect an actual meter in real life) from the person and the object in the VE. In addition, to consider how groups changed fixations over time to individual target and distractor objects, the Duration of Individual Fixations for refixations (i.e., fixation counts 3–20) were subtracted from initial fixations (fixation counts 1–2), called Change in Duration of Individual Fixations. A positive Change in Duration of Individual Fixations indicates a reduction in fixation duration in refixations. For a complete list of variables and their definitions, please see S1 Table.

To examine the effect of Military Status on manipulated cognitive load during a navigation task, visual behavior was compared outside the Math Task (before and after the Math Task) and during the Math Task (beginning with the first question and ending after the final question). Performance on the Math Task was measured by Math Score (1 point awarded for each set correctly summed, for a maximum of 3 points. The Mean Number of Fixations *per object* and Mean Dwell Time *per object* were compared between groups during and outside the Math Task. The comparison of visual attention on targets and distractors outside and during the Math Task was not conducted due to the unregulated position of the participants in the VE when the Math Task was presented. This is because the Math Task occurred at a time point and not a physical place in the world; thus, some participants could have had more opportunities to focus on a target depending on their positional location within the VE during the Math Task. Therefore, variables were averaged over all objects that were fixated on during these time points, without distinguishing between targets and distractors. The following variables were calculated and compared during and outside the Math Task: Fixation Rate (the total number of fixations per second), Object Rate (the number of unique objects fixated on in the VE per second), Blink Rate, Proportion of Fixations on Objects in the VE (as opposed to those fixations on terrain/sky), Position Velocity (meters navigated in the VE per second), Saccade Rate (number of saccades per second), Saccade Velocity (angle per second), Saccade Magnitude (the distance of the saccade in angle), and Pupil Diameter.

As noted, the Active Duty and Civilian groups were collected at two different site locations. Although the sites for data collection were similar in setup, differences existed that therefore could have impacted data collection. In examining Baseline Task data, there were a significantly larger number of unexplained dropouts with the Civilian group (17.3 ± 13.1%) compared to the Active Duty (4.6 ± 6.7%), defined as epoch of missing samples in the data that are longer in duration than the maximum blink threshold (>500 ms) (Independent-Samples Mann-Whitney U Test (MWW), *p* = 0.007). Because of these large dropouts (that were mostly in smaller continuous periods of time), several outcomes based on an occurrence per second (Fixation Rate, Saccade Rate, Object Rate, and Blink Rate) were adjusted by subtracting the unexplained dropout time from the overall time in the VE (e.g., Fixations Rate = Number of Fixations / (total time—total unexplained dropout time)). This adjustment was applied to eye tracking data from both groups and allowed us to compare across the locations while taking into consideration any setup differences. It should be noted that these dropouts could skew fixations and saccade outcomes, particularly for the Civilian group (see Discussion). However, even with dropouts, given the length of time spent in the environment and the continuous nature of the dropouts, there was a still large amount of preserved comparative data to allow for the analyses (a minimum of 900 saccades and 870 fixations per subject).

#### Data processing of the EEG data and calculation of CNN output

The neural decoding approach described in Solon et al. (2019) was used to analyze evoked responses in the EEG data during the Primary Visual Search Task [76]. The decoder, a multilayer Convolutional Neural Network (CNN), was trained using previously conducted experimental data sets to detect patterns of neural activity similar to the well-studied P300 evoked response [29, 70]. This approach was previously developed and applied for P300 signal decoding [71–73]. The CNN model used the EEGNet architecture [74], which is a compact (i.e., low number of free parameters) CNN specifically designed for EEG data. The EEGNet model was fit with 4 temporal filters, 2 spatial filters per temporal filter, and 16 separable filters (EEGNet 4-2-16), using the notation from Lawhern et al. [74]. A temporal filter length of 64 samples was used, representing 0.5 seconds of data sampled at 128 Hz, and an epoch size of 1.25 seconds. The model was trained for 150 iterations using the Adam optimizer with default parameter settings, and a minibatch size of 16 instances, optimizing a binary cross-entropy loss function [75]. There was minimal change observed in the training set cross-entropy loss beyond 150 iterations and dropout probability was set to 0.25.

Training of the CNN used the four data sets outlined in Solon et al. [76]. Each experiment was conducted with a 64-channel BioSemi Active II and each experiment was designed to investigate a P300 visual response. Each data set contained two mastoid signals, averaged, and used as reference, and were down-sampled to 128 Hz and bandpass filtered between [0.3, 50] Hz by first low-pass filtering at 50 Hz using a finite-impulse response (FIR) filter and then high-pass filtering at 0.3 Hz using another FIR filter. A median absolute deviation normalization was performed for the data from each participant prior to training or testing the CNN. Further details on how the training data was prepared is described in Solon et al. [76]. Once trained, the CNN was applied to test data one sample at a time. This convolution produced an output waveform that was time-locked to fixations in the Primary Visual Search Task. Keeping with the convention in Solon et al. [76], the time-value associated with the first sample in the epoch of data passed to the CNN is the application time. This CNN allows us to detect patterns of neural activity similar to P300 (P300-*like*), where a higher CNN output (amplitude) is associated with target acquisition and increased confidence in decision making, awareness, and engagement, allowing us to make important inferences about an individual’s SA performance.

#### Calculation of study variables with EEG data

To examine the impact of Military Status on the neural response to visual target detection, we compared the CNN outputs between the groups and between target/distractors for the Primary Visual Search Task. The first step was to z-score the CNN outputs per individual to facilitate cross-participant analysis. Next, we defined events to create time-locked repeated trials. We used the onset of a fixation as the time-locking event where T = 0 referred to the moment of fixation. A peak response at T = 0 indicates an enhanced likelihood of a P300-like response in the T = 0 to T = 1.25-second window. We use the term P300-like due to the unconstrained nature of the Primary Visual Search Task and to stay consistent with the convention of present guidance in literature [77, 78].

For the Primary Visual Search Task, CNN outputs were averaged over the window of time [–0.65, –0.20] seconds prior to the fixation onset (T = 0). This time window was based on visual inspection of the data; however, this window also produced the strongest estimates of neural activity occurring at the moment of fixation or slightly after that (e.g., CNN activity at T = –0.65 is a summary of the neural activity in the window [–0.65, 0.6] seconds). In addition to considering neural responses in this window of time, and complimentary to the Change in Duration of Individual Fixations analysis, we also compared neural responses from initial fixations and refixations on each stimulus. For both groups the change in mean CNN output response (target or nontarget) for refixations was subtracted from the mean response for initial fixations, where larger values indicate that the neural processing of stimuli occurs predominantly for the initial fixations.

CNN output comparison during and outside the Math Task was not conducted due to the relatively short duration of the Math Task. CNN output was not used to compare differences in cognitive responses between these two time periods. Therefore, analysis of cognitive activity for the Math Task was limited to pupillometry measures from the eye tracker.

### Statistical analysis

Normal distribution of the data was assessed using the Shapiro–Wilk test for normality and violations of the assumption of equal variances between the two groups was checked using the Levene’s test. All variables were interval data that passed assumptions of acceptable normality and homogeneity of variances, with the exception of Self-Reported Target Count, Gaze-Validated Targets, Math Score, and Blink Rate, which violated one or more assumptions. Due to violations of assumptions, non-parametric tests (e.g., Mann Whitney Wilcoxon [MWW] test) were used for statistical comparison for detecting group differences for Gaze-Validated Target Count, Self-Reported Target Count, and Math Score data in the Primary Visual Search Task. For data that violated normality and homogeneity of variance assumptions and required a two-way analysis, where examining both main effects and the interaction of these main effects was a priority, data transformations were applied to the data. For this reason, an arcsine transformation was applied to the Change in Individual Fixation Duration data and a log transformation was applied to the Blink Rate and Pupil Diameter data. Transformed data only were used in the statistical analysis for these variables. Parametric tests (e.g., Paired Samples T-test, Analysis of Variance [ANOVA]) were used for all other variables (and the transformed data) to compare behavior for Active Duty and Civilians.

A total of 36 participants were recruited for this study (N = 21 for the Active Duty Group and N = 16 for the Civilian group). However, some data was removed due to poor quality of the eye tracking data and/or the removal of data outliers as detailed in the following paragraphs. For a complete list outlining final participant numbers, please refer to the Study Variable List and Included Participants located in the Supplemental Information, S1 Table.

For the eye-tracking data, data for two Civilian participants and five Active Duty participants was removed from analysis due to large drops in eye-tracking data. For the Primary Visual Search Task, one Civilian and one Active Duty participant did not respond to prompting for the Self-Reported Target Count. For analysis with the Math Task and the calculation of the variable Math Score, two Active Duty participants did not respond to prompting to report summations. Also, since participants navigated at a self-selected pace and the Math Task occurred at a time point in the navigation rather than triggering at a physical location in the VE (approximately 8 minutes into the paradigm), one Civilian participant finished navigation prior to the commencement of the Math Task. Thus, this person’s data is not included for eye-tracking analysis and did not report a Math Score for the Math Task.

Outliers were those data points that were greater or less than three standard deviations from the mean and were removed prior to statistical analysis. For this reason, data was removed from analysis for two Civilian participants for the Change in Duration of Individual Fixations data, one participant’s Blink Rate data, one participant’s Duration of Individual Fixations data, and two Civilian’s Change in CNN Output data. A *p*-value of less than 0.05 was considered significant for all analysis. All statistical analysis was carried out using IBM Statistical Package for Social Science (SPSS) for Windows (Version 22, Armonk, NY: IBM Corp, Released 2013) software.

## Results

### Gaze outcomes

#### Gaze outcomes on primary visual search task: Targets vs. distractors

A Wilcoxon Signed Rank Test determined that the Active Duty group’s Self-Reported Target Count was significantly higher than the Civilian group (Z = 2.21, *p* = 0.027). The Active Duty group reported a median 15 (interquartile range [IQR] = 15, 16) targets compared to 14 (IQR = 12, 15) targets for the Civilian group. There was no significant difference between Self-Reported Target Count and the number of Gaze-Validated Targets for either the Civilian (Z = –0.54, *p* = 0.590) or the Active Duty groups (Z = –0.95, *p* = 0.340).

**Fig 3:**
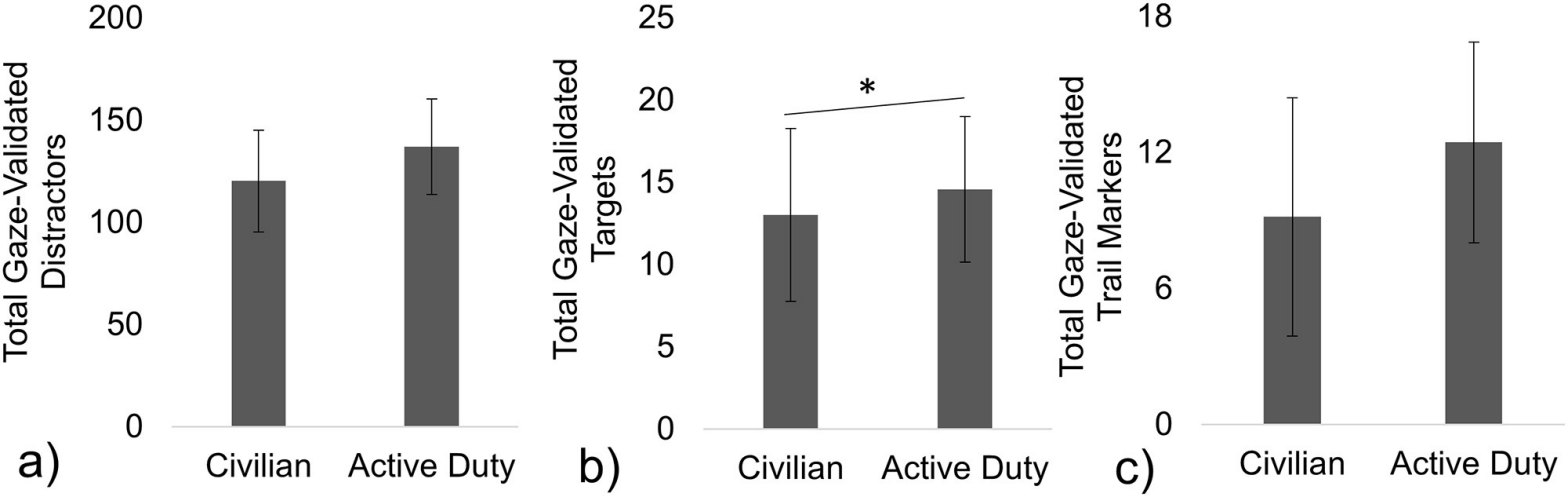
Total Gaze-Validated objects in the VE. The Active Duty group saw significantly greater numbers of Gaze-Validated Targets (b). The two groups fixated on a similar number of Total Gaze-Validated Distractors (a) and Trail Markers (c). Mean ± SDs (error bars) are shown on the graph.

Separate independent T-Tests found no significant group differences between the Total Gaze-Validated Distractor objects (Fig 3a, *t* = –1.83, *p* = 0.079) or the Total Gaze-Validated Trail Markers (Fig 3c, *t* = –1.86, *p* = 0.076). However, a MWW test found that the Active Duty group fixated on a significantly greater median number of Total Gaze-Validated Targets, 15 (IQR = 14, 15), compared to Civilians, 14 (IQR = 11, 15) (Fig 3b, Z = 2.31, *p* = 0.032). The median Total Path Distance traveled in the VE for the Active Duty was 2701 (IQR = 2665.71, 2818.80) meters and compared similarly to the Civilian group’s median of 2681 (IQR = 2653.61, 2865.14) meters (MWW, Z = 0.18, *p* = 0.88). Also, the median Total Time spent in the VE, 805 (IQR = 775.93, 890.25) seconds and 795 (IQR = 774.39, 852.87) seconds was similar for the Active Duty and Civilian groups, respectively (Z = 0.26, *p* = 0.81).

**Fig 4:**
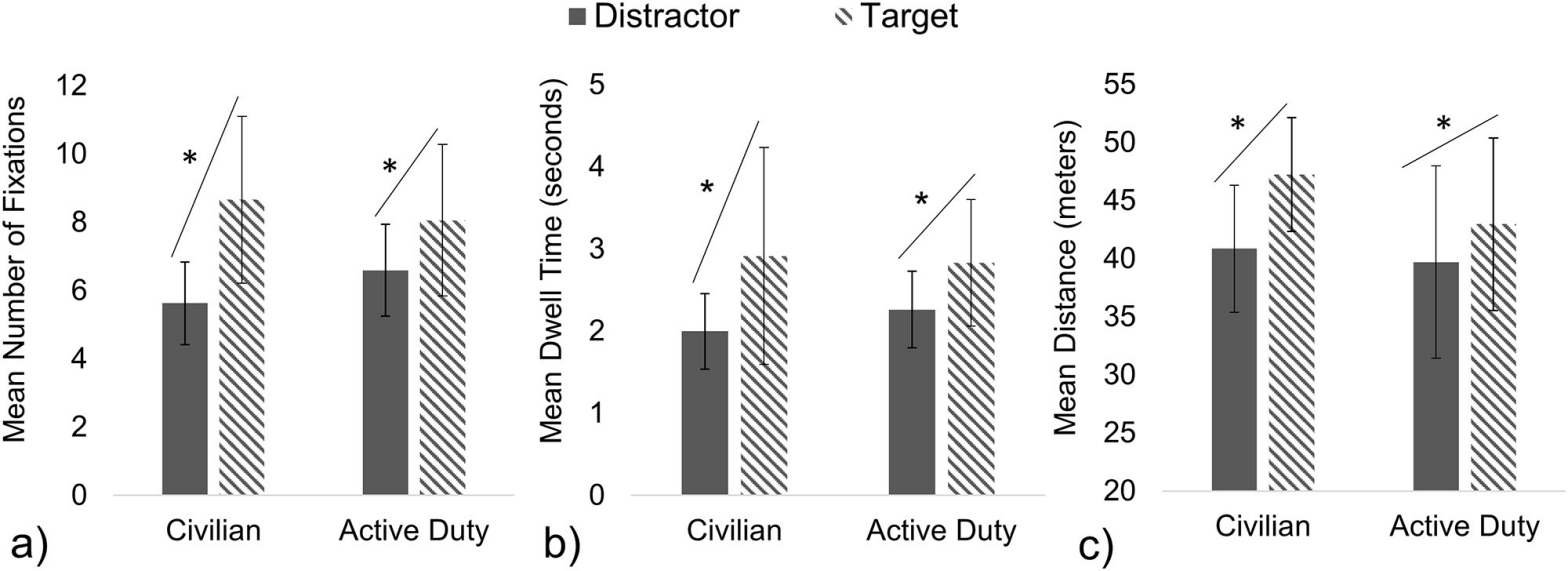
Number of Fixations, Dwell Time, and Mean Distance to targets and distractors. There were no significant differences between groups, and both groups significantly increased the Mean Number of Fixations (a), Mean Dwell Time (b), and Mean Distance (c) similarly, for targets compared to distractors.

A two-way Multivariate Analysis of Variance (MANOVA) determined that the Mean Number of Fixations and Mean Dwell Time (Fig 4a and 4b) was statistically dependent upon the Object (target, distractor) (F (2, 26) = 21.60, *p* < 0.000) but not statistically dependent on the Military Status (F (2, 26) = 0.60, *p* = 0.557) or the interaction of Object and Military Status (F (2, 26) = 3.36, *p* = 0.050). Follow-up univariate analysis determined that regardless of Military Status, both groups significantly increased the Mean Number of Fixations (F (1, 27) = 41.13, *p* < 0.000) and Mean Dwell Time (F (1, 27) = 13.41, *p* < 0.000) on targets compared to distractors. An additional two-way ANOVA found that Mean Distance (Fig 4c) was significantly dependent upon Object (F (1, 27) = 27.73, *p* < 0.000), but was not significantly impacted by Military Status (F (1, 27) = 1.31, *p* = 0.263) or the interaction between Military Status and Object (F (1, 27) = 2.88, *p* = 0.101). Both groups fixated on targets at a closer distance on average compared to distractors.

#### Gaze outcomes on Primary Visual Search Task: Habituation to targets and distractors

**Fig 5:**
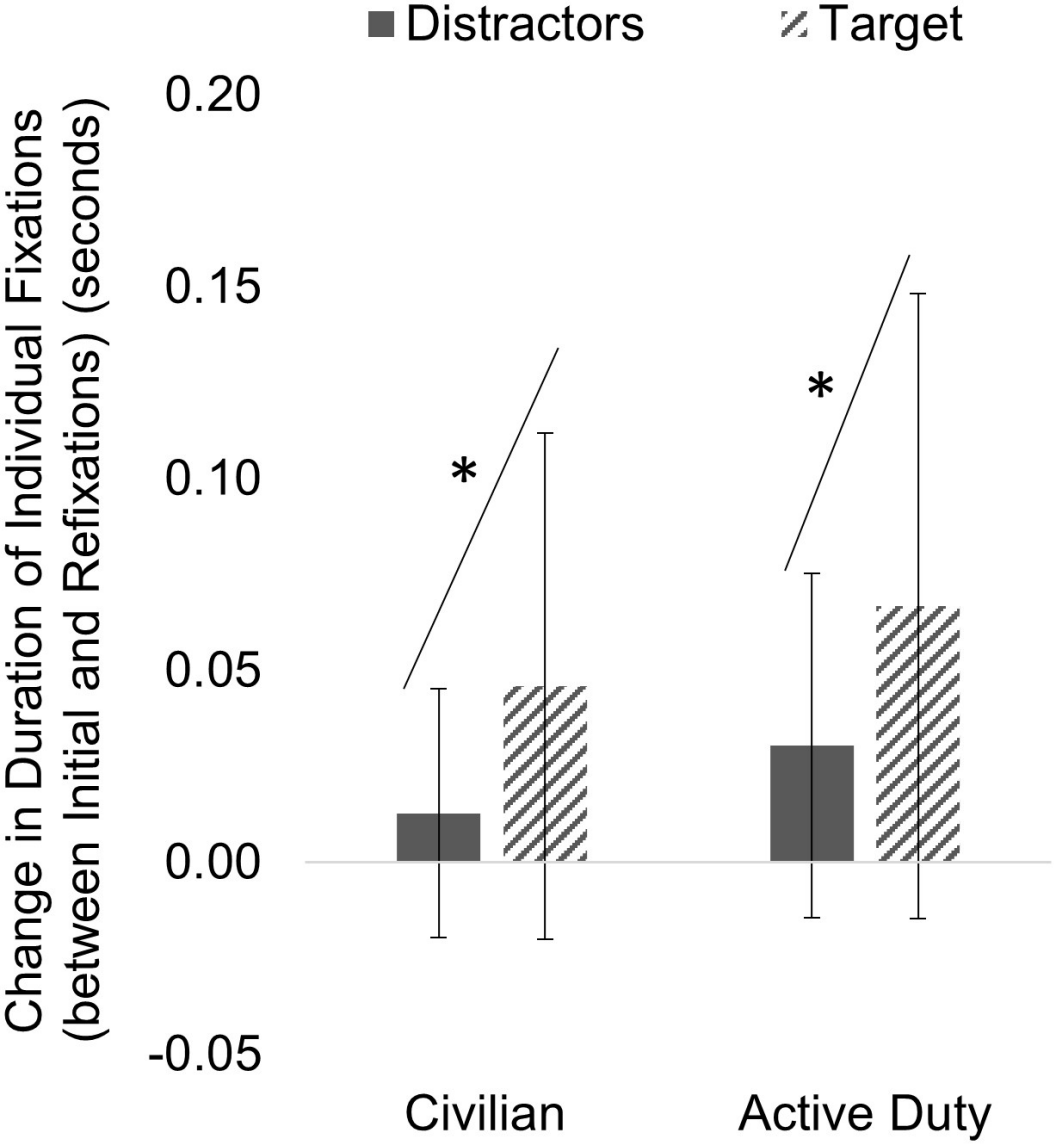
Habituation of fixation duration to targets and distractors. There were no group differences in Change in Duration of Individual Fixations between the Initial Fixations (fixations 1–2) and Refixations (fixations >2). There was a greater Change in Duration of Individual Fixations for the targets compared to the distractors for both groups.

Next, we divided the data from each participant into Initial Fixations (fixations 1–2) and Refixations (>2 subsequent fixations) on the same object. Then we examined the Individual Fixation Duration for refixations (i.e., fixations 3–20) subtracted from the CNN Output for initial fixations (i.e., fixations 1 and 2) on each object, called Change in Duration of Individual Fixations (Fig 5). A positive change indicated the average Duration of Individual Fixations for the initial fixations was longer compared to refixations. A mixed 2 × 2 ANOVA found that Change in Duration of Individual Fixation was significantly different between objects (F (1, 56) = 4.50, *p* = 0.039) but not for group (F (1, 56) = 0.72, *p* = 0.399) or the interaction of group and object (F (1, 56) = 0.17, *p* = 0.681). Both Civilian and Active Duty individuals reduced individual fixation durations when refixating on targets and distractors. For both groups, there was a greater reduction (positive change) in fixation time for targets compared to distractors when looking at the difference between initial and refixations.

#### Gaze outcomes on secondary Math Task: The impact of manipulated cognitive load

The Civilian group overall had a significantly higher median Math Score compared to the Active Duty group (MWW, Z = –2.54, *p* = 0.011). The Civilian group, on average, scored 3 (IQR = 2, 3) on the Math Task compared to a median score of 2 (IQR = 2, 2) for the Active Duty group.

**Fig 6:**
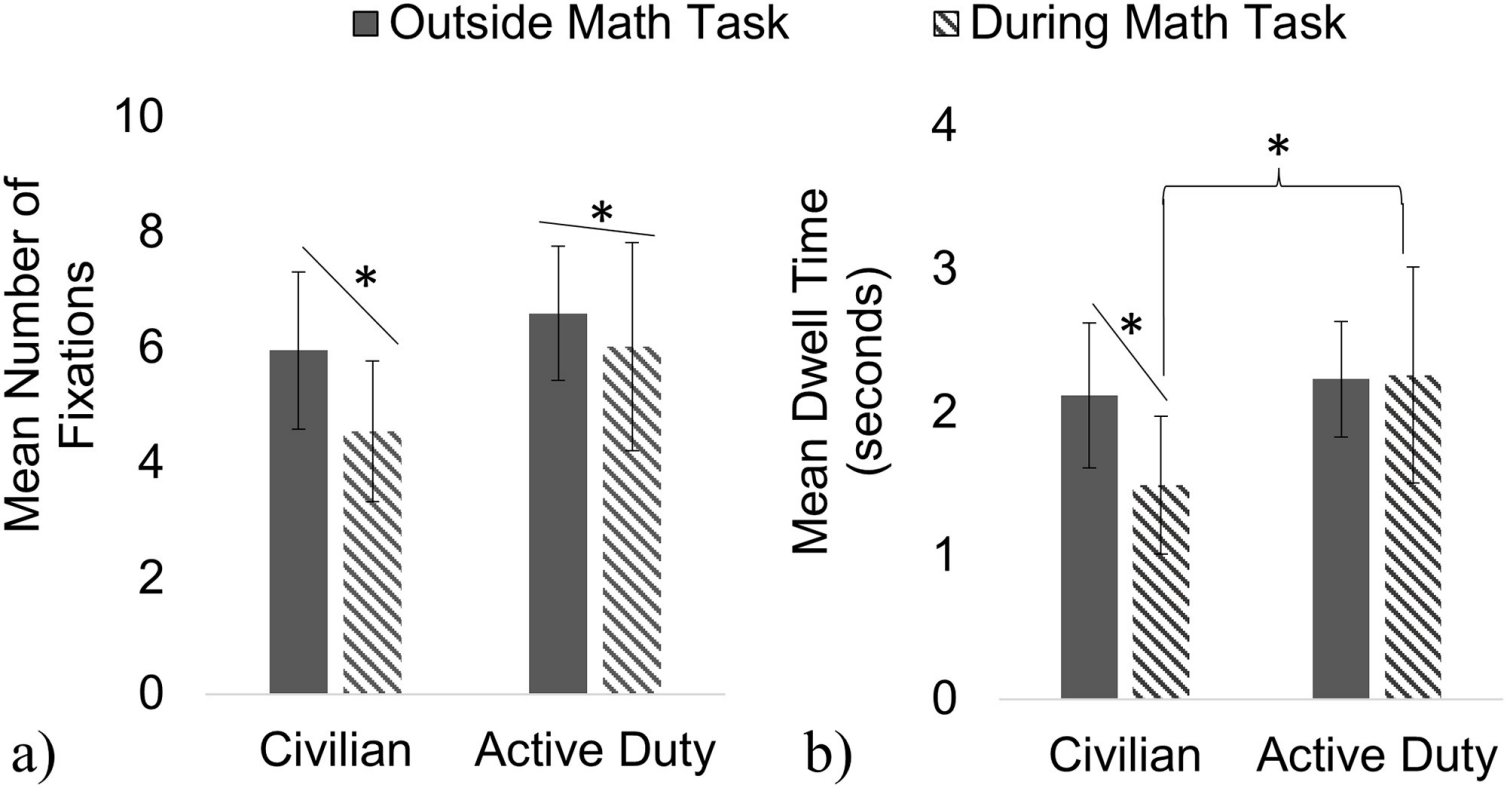
Number of Fixations and Dwell Time on objects during Math Task. Both groups similarly decreased Mean Number of Fixations on objects during the Math Task compared to outside the Math Task (a). The Civilian group significantly decreased Mean Dwell Time on each object during the Math Task compared to outside the Math Task, whereas the Active Duty group did not significantly differ in Mean Dwell Time on Objects during the Math Task (b).

To capture how military status impacted participants’ visual exploration of the environment during manipulated cognitive load, a mixed-design MANOVA assessed how Military Status impacted the Mean Number of Fixations and Mean Dwell Time on *objects* in the environment when cognitive load was manipulated with a Math Task (Fig 6a and 6b). Overall, the Math Task (F (2, 25) = 9.62, *p* = 0.001) and the interaction of Military Status and Math Task (F (2, 25) = 4.87, *p* = 0.016) were significant. Both the Active Duty and Civilian groups significantly decreased the Mean Number of Fixations during the Math Task compared to outside the Math Task (F (1, 26) = 19.10, *p <* 0.000). A follow-up Univariate ANOVA determined that the interaction of Military Status and Math Task was significant for Mean Dwell Time (F (1, 26) = 9.33, *p* = 0.005) but not the Mean Number of Fixations (F (1, 26) = 3.28, *p* = 0.082). The Civilian group significantly decreased Mean Dwell Time on objects while navigating during the Math Task compared to outside the Math Task (*p* < 0.01, Tukey Post-hoc), whereas the Active Group did not significantly adapt Mean Dwell Time (*p* > 0.05, Tukey Post-hoc).

A two-way mixed ANOVA found that changes in pupil diameter outside of and during the Math Task was similar for both groups (F (1, 26) = 0.40, *p* = 0.531). When investigating dilation over the course of the entirety of the Math Task, both groups significantly increased mean pupil diameter for during the Math Task compared to outside the Math Task (F (1, 26) = 57.37, *p* < 0.001). The interaction between group and Math Task for pupil diameter was not significant (F (1, 26) = 3.05, *p* = 0.093).

**Fig 7:**
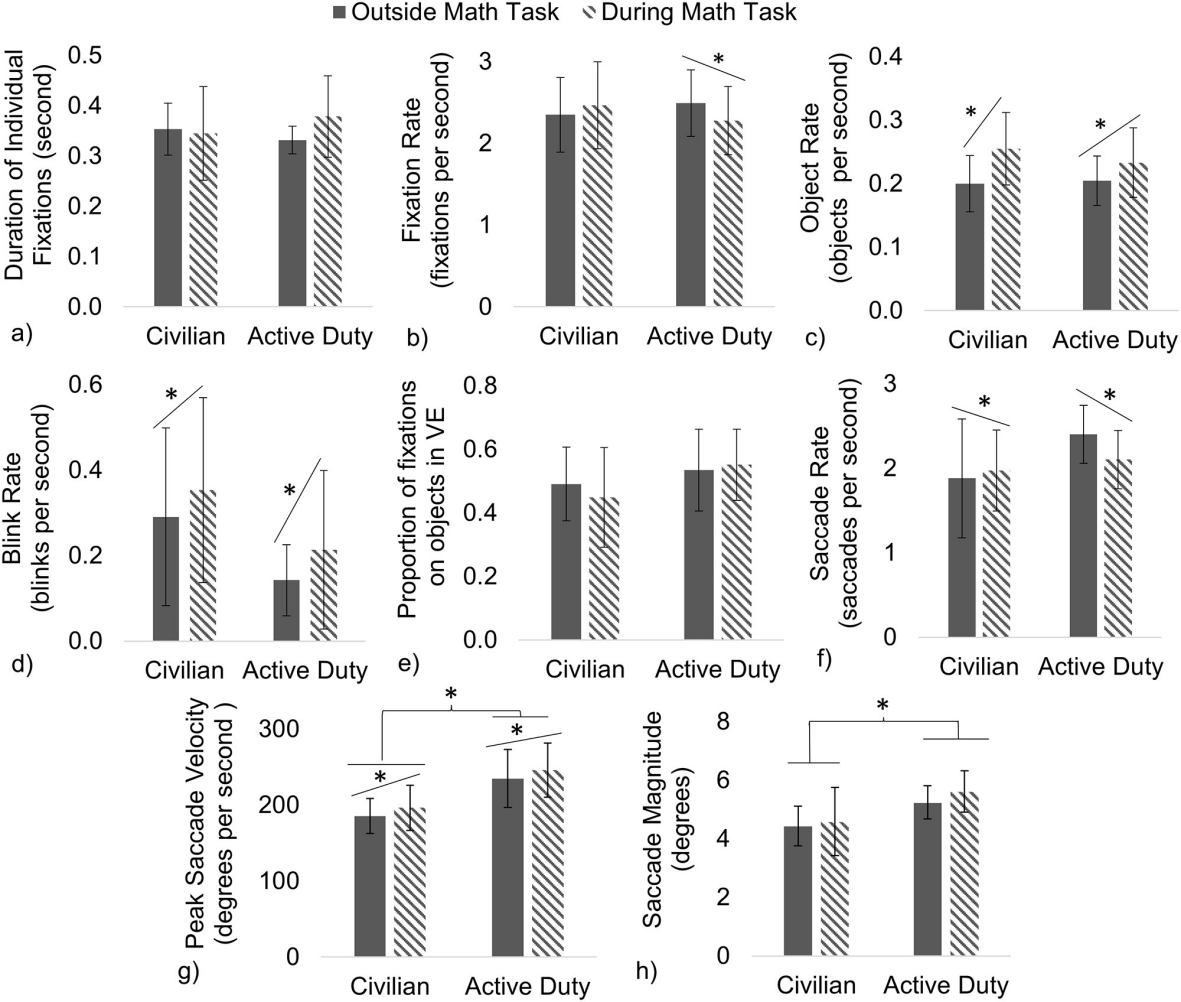
Math Task fixation, blink, and saccade outcomes. With manipulated cognitive load, both groups significantly increased Object Rate (c), Blink Rate (d), and Peak Saccade Velocity (h) and decreased their Saccade Rate (f), while only the Active Duty group decreased their Fixation Rate (b) with the Math Task. The Active Duty group differed significantly from Civilians in their visual scanning behavior with increased Saccade Velocity (g) and Saccade Magnitude (h) compared to the Civilian group. There were no significant impacts of group or Math Task for the Duration of Individual Fixations (a) or the proportion of fixations on objects in the VE (as opposed to on Terrain/Sky) (e).

Separate two-way mixed ANOVAs determined the effect of Military Status and Math Task on the Duration of Individual Fixations, Fixation Rate, Object Rate, the Proportion of Fixations on Objects in VE (vs. Terrain/Sky), Saccade Rate, Peak Saccade Velocity, Saccade Magnitude, Blink Rate, and Position Velocity (Fig 7). There was a significant main effect of the Math Task where both groups decreased their Saccade Rate (Fig 7f) and Position Velocity (not shown in Fig 7) while increasing their Object Rate (Fig 7c), Blink Rate (Fig 7d), and Saccade Velocity (Fig 7g), on average, when cognitive load was manipulated with the Math Task. There was also a significant effect of Military Status where the Active Duty group, overall, scanned the VE with significantly increased Peak Saccade Velocity (Fig 7g) and Saccade Magnitude (Fig 7h) compared to the Civilian group. The interaction between Military Status and the Math Task was only significant for Fixation Rate (Fig 7b), where the Active Duty group significantly decreased their Fixation Rate during the Math Task and there was no significant change in Fixation Rate observed for the Civilian group. There was no significant impact of manipulated cognitive load or group for the Duration of Individual Fixations (Fig 7a) or the Proportion of Fixations on Objects in the VE (Fig 7e). All other comparisons were not significant. A comprehensive table detailing all statistical outcomes for the impact of Military Status and manipulated cognitive load with the Math Task is included in the S2 Table.

### EEG outcomes

#### EEG outcomes: CNN Output (measuring P300-like response) to stimuli in the Primary Visual Search Task

**Fig 8:**
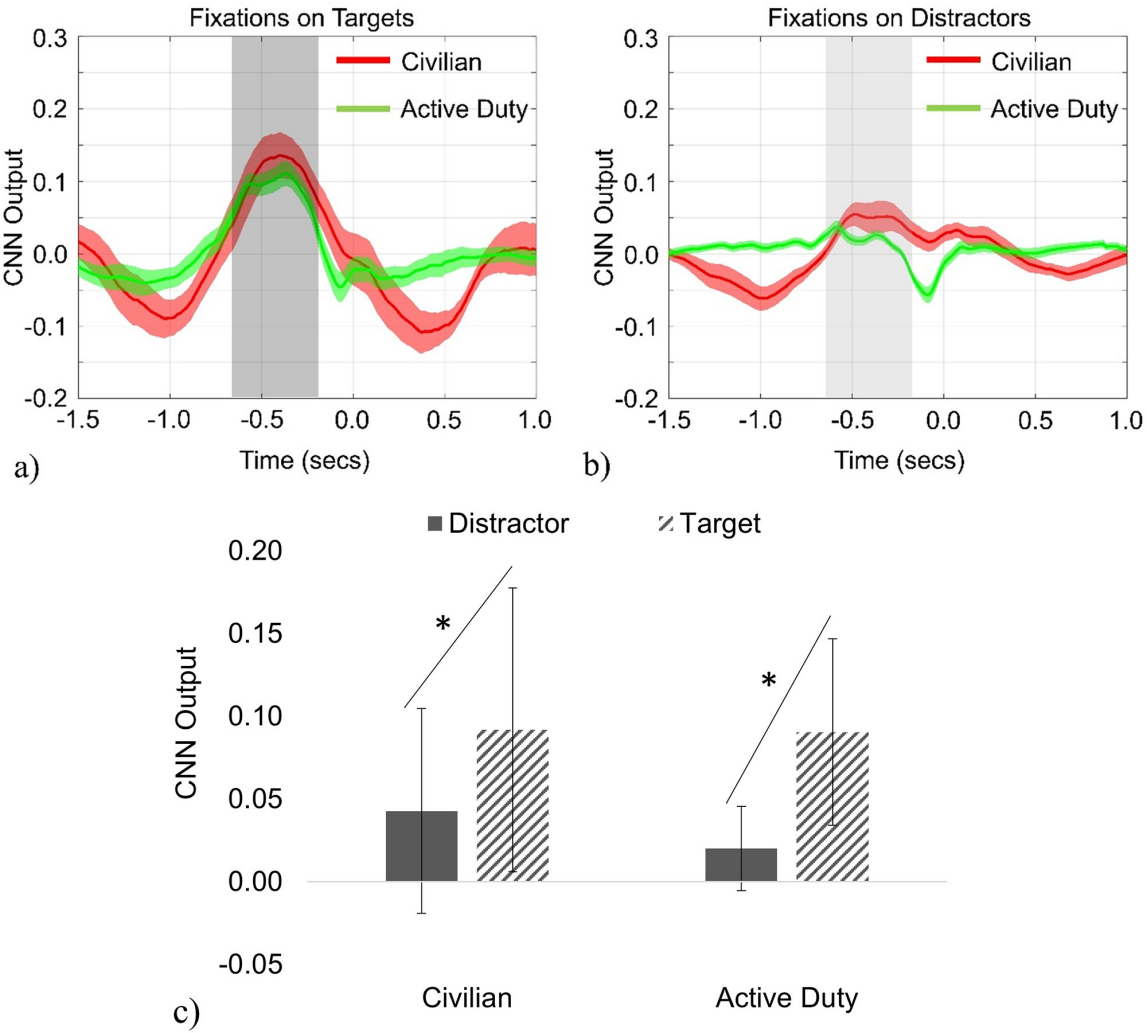
CNN Output. Mean CNN Output for the primary free-viewing task where T = 0 is the moment of fixation. CNN Output (response profiles) were similar for both Civilians and Active Duty for all fixations on targets (a) and distractors (b). Both groups similarly decreased CNN Output for distractors compared to targets (c).

In the Primary Visual Search Task, mean CNN Output was compared between fixations on targets and distractors visually (Fig 8a and 8b) and mean CNN Output was compared between groups and Objects (targets, distractors) with a Repeated Measures ANOVA. When considering all fixations on targets or distractors (Fig 8a and 8b), both groups exhibited similar activity that peaked in the window [–0.65, –0.2] seconds relative to fixation onset (T = 0). For Mean CNN Output, there was no main effect of group (F (1, 72) = 0.41, *p* = 0.525) but there was a main effect of Object (F (1, 72) = 16.35, *p* < 0.001). Both groups exhibited a similar significant increase in likelihood in P300-like activity around the moment of fixation for targets compared to distractors (Fig 8c, F (1, 72) = 0.96, *p* = 0.332 for the interaction of group and object).

**Fig 9:**
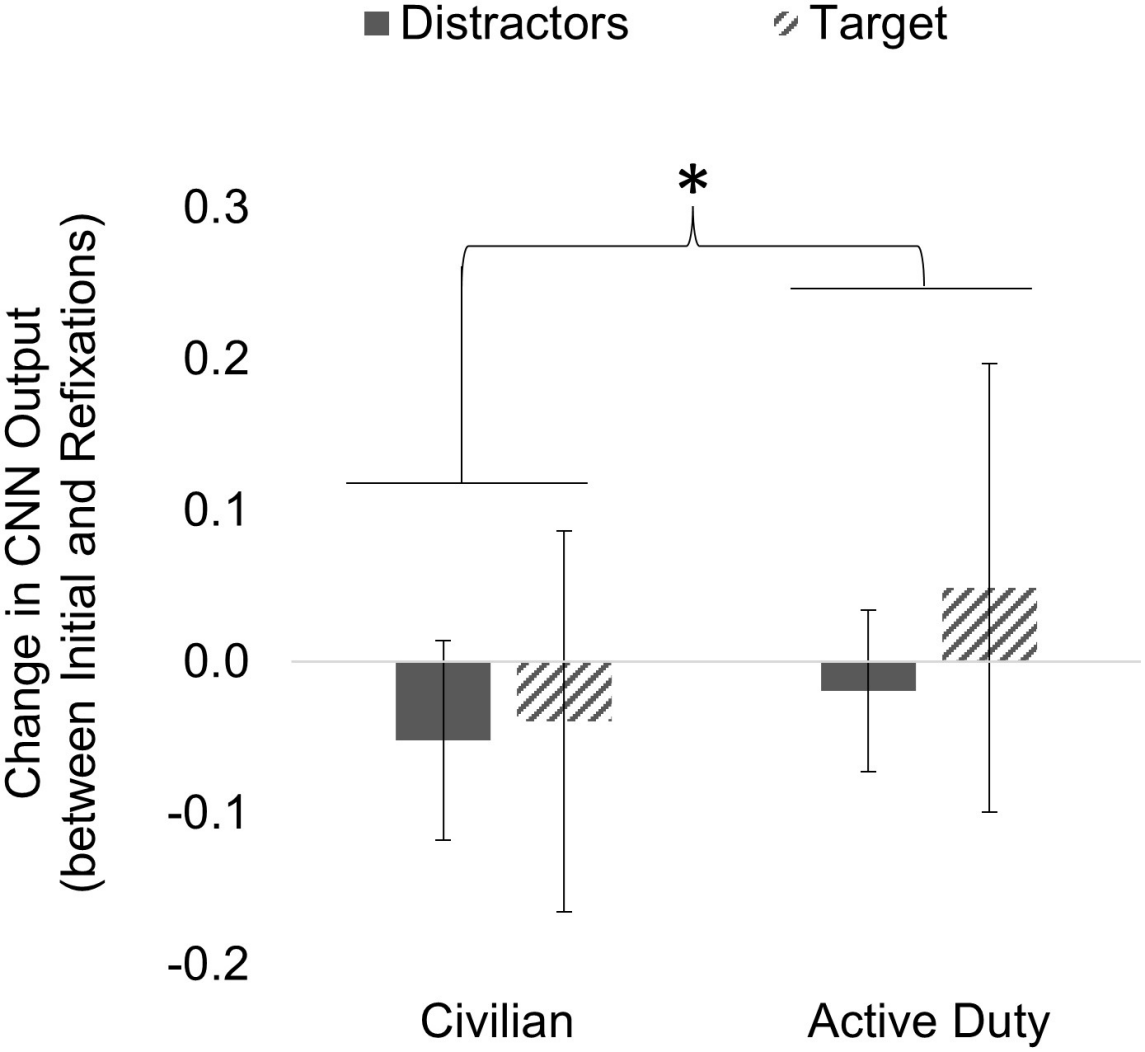
Change in CNN Output. Change in CNN Output, defined as the CNN Output of refixations (fixations 3–20) subtracted from CNN Output of initial fixations (fixations 1–2), was significantly different between Civilians and Active Duty groups overall.

For both groups, the CNN Output for refixations (i.e., fixations 3–20) was subtracted from the CNN Output for initial fixations (i.e., fixations 1 and 2) on each object fixated on, called Change in CNN Output (Fig 9). Larger values indicated that the neural processing of stimuli occurs predominantly for the initial fixations. A mixed 2 × 2 ANOVA found that Change of CNN Output was significantly different between groups (F (1, 71) = 5.52, *p* = 0.022) but not for object (F (1, 71) = 2.47, *p* = 0.121) or the interaction of group and object (F (1, 71) = 1.15, *p* = 0.287). Civilian participants exhibit a negative change in CNN outputs, indicating more neural activity occurring for refixations versus initial fixations. The Active Duty group exhibits the opposite trend with refixations producing lower CNN outputs.

## Discussion

### Primary Visual Search Task performance and eye behavior

Overall, there were differences in how the Active Duty and Civilian individuals performed in the navigational task in the desktop VE. In terms of performance on the Primary Visual Search Task and the Math Task, the Active Duty group appeared to perform better than the Civilian group in terms of finding targets in the Primary Visual Search Task (higher/closer to correct number Self-Report Target Count) but had lower performance compared to the Civilian group in the Math Task (lower Math Score). In terms of gaze behavior for the Primary Visual Search Task, there were significant differences between Active Duty and Civilians, but these were limited mainly to the visual scanning behavior. Visual scanning differences were observed regardless of workload (in the introduction of the secondary Math Task). We found that although the two groups did not differ in the average Number of Fixations or Dwell Time on targets and distractors, the military service members did scan the environment with faster Peak Saccade Velocities and a greater average Saccade Magnitude. This behavior has previously been found with expert’s visual searches [39] and could be indicative of superior skill in scanning peripheral regions [41]. Visual search is a task that requires a continual, broad scanning of the environment while maximizing coverage and finding targets. It may be possible that individuals with experience in real-world target detection may demonstrate more visual scanning behaviors that indicates anticipation for future target detection and minimizes target positional uncertainty. Another possibility is that experts may exhibit this type of scanning behavior because their expertise allows them to have more cognitive resources available to process periphery visual cues as opposed to just resourcing visual cues closer to the foveal (central) eye region [40, 41]. It may be possible that those individuals with experience in real-world target detection and navigation (e.g., military), would have increased focus on objects in the periphery (further from the path), as evidenced by the increased average Saccade Magnitude. Increased Peak Saccade Velocity is not controlled voluntarily [79, 80], has been linked to increased arousal during naturalistic tasks [25], and is thought to be a better indication of underlying cognitive activity over other gaze behavior [81]. Although other fixation metrics (average number of fixations and dwell time) could have been underestimated due to the larger dropouts (>500 ms) in the data, differences in saccade velocities and saccade magnitudes are interesting as this data (saccade durations range from 12–100 ms [65, 80]) is assumed to be relatively preserved even with the differences in dropouts. In general, although there was very little difference in the fixation data between the groups for the Primary Visual Search Task (e.g., group differences in average Number of Fixations, Dwell Time, and Change in Duration of Individual Fixations for initial and refixations on targets and distractors), the Active Duty group’s scanning behavior (increased Peak Saccade Velocity and increased Saccade Magnitude) could be indicative of increased SA.

The Active Duty group appeared to have a higher performance on reporting the correct number of targets and seeing targets in the Primary Visual Search Task, but these results should be interpreted cautiously. The Active Duty group saw significantly more targets (with at least one qualifying eye fixation) compared to Civilians. However, this difference could be attributed to the larger number of Unexplained Dropouts present in the Civilian data compared to the Active Duty data, enabling the eye tracker to capture more qualifying fixations on objects in the environment for the Active Duty group. Fewer Unexplained Dropouts with the Active Duty group could be evidence that the group tended to better maintain positional alignment with the eye tracker compared to the Civilian group, or it could be attributed to setup differences between the site locations. Although interpretation of these eye-tracking findings is limited, there is some supporting evidence for higher performance for the Active Duty group in the Primary Visual Search Task, in the Self-Report Target Count data. The Active Duty group, on average, reported seeing closer to the correct number of targets, compared to the Civilian group who, on average, significantly underreported the number of targets in the VE. However, there was no difference between the number of targets counted by individuals and the number of those identified by observed gaze direction for either group. This showed that although the Active Duty group may have identified more targets overall, both groups were equally good at keeping track of counting the targets visually seen during the navigation task.

### Primary Visual Search Task CNN Output (EEG)

While we observed minimal differences in the measured ocular fixation behavior (i.e., number of fixations, fixation duration, change in individual fixation duration between initial and refixations), we did observe differences “under the hood,” in the neural response. Specifically, we found group differences when examining CNN Output differences in initial fixations and refixations. These differences between initial and refixations were not found when examining the eye-tracking behavior with a similar comparison—the Change in Duration of Individual Fixations. In the aggregate (considering all fixations), both groups produced a similar increase in CNN Output (response profiles) for target objects compared to distractor objects. This appears to confirm previous work showing that an increase in CNN Output indicates the probability of a P300-like response and is associated with target acquisition. There were significant group differences when CNN Output was examined closer by examining Change of CNN Output between initial fixations and refixations. Overall, the Change of CNN Output for the Active Duty group was significantly more positive than the Civilian group, who exhibited an overall negative Change in CNN Output. It appears that the Civilian group exhibited a mean Change of CNN Output in the negative direction, indicating more neural activity occurring for refixations compared to initial fixations. The opposite pattern was found with the Active Duty group, who were more heavily localized to initial fixations, with refixations producing less neural activity. Although the object was not significant, nor was the interaction of group and object, it appears that the group difference between Active Duty and Civilians was heavily due to the difference in Change of CNN Output for target objects. The Active Duty group displayed an average Change of CNN Output that was positive (indicating more neural activity occurring for initial fixations vs. refixations) for targets, compared to the negative change response (indicating more neural activity occurring for refixations vs. initial fixations) for the Active Duty group on distractor objects, and the Civilian group on both targets and distractors. The results indicate that although overall cognitive processing is similar between groups, the Active Duty group devoted less neural resources reprocessing to VE objects in general, indicating potentially a more cognitive resource-efficient search strategy than the Civilian group.

### Group differences in allocation of attentional resources with manipulated cognitive load

When cognitive load was increased with an auditory Math Task, both groups increased alertness but differed in how they divided attentional resources between the two tasks. In general, when an individual is performing more than one task at a time, a decrement in performance is often observed and is generally thought to be due to limitations in attentional resources [45]. Therefore, we expected the groups would sacrifice (or shift) attentional resources to either the Primary Visual Search Task or the secondary Math Task. Both groups showed equal evidence of increased cognitive processing via increased pupil dilation [49, 50, 52, 54], and both groups demonstrated increased levels of arousal via increases in Peak Saccade Velocity [25]. However, the remaining eye behavior appears to unveil a difference in how the two groups split attentional resources between the two tasks. For instance, although both groups decreased the average Number of Fixations per object in the VE during the Math Task, only Civilians simultaneously decreased Dwell Time on objects in the VE. In contrast, the Active Duty group appeared to maintain average Dwell Time on objects in the VE and decrease their average Fixation Rate. This decrease in Fixation Rate was potentially accomplished through the tendency of the Active Duty group to increase the Duration of Individual Fixations by an average of 47 ms (compared to 8 ms for Civilians). In addition, the Active Duty group performed worse overall in the Math Task (decreased Math Score) compared to the Civilians. This, in conjunction with their differences in eye behavior and the Self-Report Target Count data, lends to the theory that the Active Duty group potentially allocated more attentional resources (maintained SA) to the Primary Visual Search Task over the secondary, auditory Math Task compared to Civilians.

The behavior of a military population prioritizing primary study tasks over an auditory secondary Math Task could be due to task design. The secondary task was not related to the Primary Visual Search Task and the Active Duty group appeared to prioritize (maintained SA) on the main visual task to a greater degree than the Civilian group. In addition, the relative time spent completing the secondary tasks was relatively short and occurred while participants were well into their primary visual search tasking (~8 min mark), which may have inadvertently provided the Active Duty participants with the perception they should prioritize the main primary visual search task regardless of instructions. As a reminder, we did not directly measure performance on the primary task during the secondary Math Task due to this imbalance in relative durations of time and the design of how the Math Task was triggered in the VE. Interestingly, a similar behavior was observed in a previous study looking at workload and shooting performance with Marines. When workload was increased, Marines maintained shooting performance but decreased performance on a secondary (auditory) Math Task and SA memory recall task [47]. Therefore, we may be observing a similar allocation of attentional resources for the Active Duty group. It should also be noted that the Active Duty group did not appear to completely reject attending to the secondary Math Task, as both groups significantly increased Blink Rate, indicating a shift of cognitive processing to the auditory task to some degree [53]. A future study with a more task-relevant secondary task could yield more direct conclusions to how manipulating cognitive load impacts military personnel’s SA. Such a design should also consider a closer balance in the duration of time between cognitive load manipulations and a consistent location trigger of the manipulation (between subjects) to allow for comparison of target and distractor specific fixation data. In their totality, these results do indicate that the Active Duty and Civilian groups differed in their fixation and visual scanning behavior when cognitive load was manipulated with the additional simultaneous auditory Math Task. These results also demonstrate how important it is to consider the population, particularly a military one, when designing a secondary cognitive load task.

### Study limitations

One large limitation of this study is that data was collected at two different locations. Civilians were recruited and tested in one location (LA) and Active Duty members recruited and tested in another location (JBSA). The data collection for the Civilian group was collected in an enclosed chamber, where there was not a direct line of sight between researchers and participants. In contrast, the Active Duty group was placed in a semi-transparent partition where observations and adjustments from experimenters were more likely to occur. It is possible the added line of sight alone caused the Active Duty population to naturally be more vigilant and situationally aware during the testing compared to their Civilian counterparts. This may explain the reduction in eye drop-out data if, for example, those data collectors were able to more frequently remind participants to maintain their posture and orientation when interacting in the VE. The data collectors themselves were also different, which may have introduced nuances in how the study was carried out. In an effort to mitigate experimenter–participant interaction differences, standard operating procedures in administering the experiment were identical at both sites. Although staff at one location trained and maintained constant communication with data collection staff at the other location, differences in testing personnel and location could have influenced differences found between the populations.

While the current study was originally designed to demonstrate capabilities of our VE and system to detect neurophysiological differences when targets are visually acquired (as opposed to distractors), both Civilians and Active Duty participants were recruited for the study, allowing us to draw preliminary and cursory comparisons of these two groups in terms of differences in visual processing. Thus, our capabilities to speculate on underlying mechanisms explaining group differences is limited. To attempt an explanation, the most likely reason seems to be that these two populations are approaching and performing the visual search task in a behaviorally and cognitively different way. Whether differences in eye movement and neural activity are the result of 1) intense training for SA in the Active Duty population, 2) minor differences in how the task instructions were relayed, or understood, between the groups, or 3) some other factor, is beyond the scope of the current analysis. However, that these differences exist is intriguing and implies that if scientific research is to be applied to Active Duty populations, more focus should go into these populations during the initial design phases of the scientific process. Since the Active Duty group did not appear to shift attention toward the secondary unrelated task, manipulating cognitive load (e.g. increasing cognitive load) and challenging SA via the environment itself could more accurately capture how military training impacts attentional resources. Such an environment could include threatening and nonthreatening neutral and military environments [82] and be more applicable to the type of multitasking and environment that military training is targeted toward. Regardless, even with the neutral nature of the current study stimuli and design, our findings suggest that the Active Duty group is a unique population and behaves differently than the Civilian group when searching for targets.

## Conclusion

Research focused on understanding the warfighter and how we can assist them to better meet mission goals often starts with a more readily available Civilian population. Given our findings that significant differences exist between these two populations, it is critical to understand how generalizable results collected with only a Civilian sample may be to the warfighter. This paper thus provides insight illuminating differences between a Civilian and Active Duty population regarding visual scanning behavior while manipulating cognitive load. We report differences in performance, eye tracking, and neural responses during a visual search task with an added auditory math component to modulate cognitive load. Results show there are fundamental differences in how the two groups explored the environment and operated under manipulated workload. The Active Duty group reported seeing a significantly greater number of targets in the VE and conducted the Primary Visual Search Task with overall significantly greater peak saccade velocities compared to Civilians, indicating an increased level of arousal and SA in the main study task. Furthermore, increased peak velocities for the Active Duty group were accompanied by greater average saccade magnitudes, a movement behavior linked with expert visual search strategies. Overall performance on the Math Task was lower for the Active Duty group compared to the Civilian group. Both groups exhibited similar patterns of eye behavior that is reflective of increased underlying cognitive activity. Both groups increased pupil dilation, indicating increased cognitive processing, and increased peak saccade velocities, indicating increased arousal. Both groups increased blink rate, indicating a shift in the allocation of attentional resources from visual processing to auditory processing. However, differences in eye-movement behavior (decreasing fixation rate and maintaining Mean Dwell time on objects), provides evidence that the Active Duty prioritized attentional resources to the visual task over the auditory Math Task, accounting for reduced performance on the Math Task for the Active Duty group. Unseen when examining initial and refixations by eye behavior alone (individual duration of fixations), neural analyses results suggest that the Active Duty group was cognitively processing objects differently than their Civilian counterparts. The Active Duty individuals devoted cognitive resources for the initial processing these of VE objects (early fixations) but reduced attentional resources devoted to these objects in later fixations. In contrast, the Civilian group appeared to increase cognitive resources in refixations. Given these findings and the implication that Active Duty and Civilians did not respond or behave the same way during tasks, it is important to consider carefully which population is appropriate to include in studies, and for studies using both, to examine whether differences exist and the implication of these differences on the generalizability of results. Furthermore, this study provides concurrent evidence of physiological signals (e.g., eye gaze and brain) as viable resources for assessing SA. Eye-gaze behavior is an especially valuable resource for military populations as it may be opportunistically or passively sensed without adding cognitive or physical burden to the Soldier. While less ruggedizable or available in the field, EEG, and in particular the CNN Output, provided a clear P300-like signal in a virtual naturalistic environment allowing us to examine the cognitive mechanisms underlying SA. This can be leveraged in future laboratory tests to glean important information about SA.

## Supporting information

S1 Table Study Variable List and Included Participants. Each outcome definition, the comparative analysis they are included in, and the final participant numbers in that analysis following exclusion due to either large dropouts in eye-tracking data, missing data, or outliers.
(DOCX)

S2 Table F-statistic details for eye-tracking outcomes during the Math Task. The detailed F-statistics for the separate ANOVAs used to determine the effect of Military Status and Math Task and their associated degrees of freedom and *p*-values.
(DOCX)
