# Supplementary material for: Evidence of elevated situational awareness for active duty soldiers during navigation of a virtual environment
Source: PLoS One. 2024 May 10;19(5):e0298867. doi: 10.1371/journal.pone.0298867 (PMC11086823; doi:10.1371/journal.pone.0298867)
Supplement: S1 Table — Each outcome definition, the comparative analysis they are included in, and the final participant numbers in that analysis following exclusion due to either large dropouts in eye-tracking data, missing data, or outliers. (DOCX) [file pone.0298867.s001.docx]

| **Study Variables** | | | | **Final Participant Numbers** | |
| --- | --- | --- | --- | --- | --- |
| **Variable** | **Definition** | **Primary Visual Search Task** | **Cognitive Load**  **Manipulation Task** | **Active**  **Duty**  **Total**  **N = 21** | **Civilian**  **Total**  **N = 15** |
| **Self-Reported Target Count** | Participant reported total count of targets | X | . . . | 20 | 14 |
| **Gaze-Validated Targets, Distractors, Trail Markers** | Total number of targets/distractors/trail markers having at least one qualifying fixation | X | . . . | 16 | 13 |
| **Total Path Distance** | Total distance (meters) avatar traveled in VE | X | . . . | 16 | 13 |
| **Total Time** | Total time (seconds) avatar spent in VE | X | . . . | 16 | 13 |
| **Mean Number of Fixations** | Total number of qualifying fixations per each object | X | X | 16 | 13 |
| **Mean Dwell Time** | Total duration of fixations per each object | X | X | 16 | 13 |
| **Mean Distance** | Average distance from avatar to object in VE | X | . . . | 16 | 13 |
| **Change in Duration of Individual Fixations** | Average duration of refixations subtracted from mean duration of initial fixations on each object | X | . . . | 16 | 11 |
| **Math Score** | Total math score; 1 point awarded for each correct set summation, maximum of 3 points | . . . | X | 20 | 14 |
| **Mean Duration of Individual Fixations** | Average of all individual fixations across all objects (targets and distractors) in the VE | . . . | X | 15 | 11 |
| **Pupil Diameter** | Average size of the pupil | . . . | X | 16 | 12 |
| **Fixation Rate** | Summation of all fixations during Math Task (or outside Math Task) divided by duration of that time period | . . . | X | 16 | 12 |
| **Object Rate** | Total number of distinct objects fixated on per unit time | . . . | X | 16 | 12 |
| **Proportion of Fixations on Objects in the VE** | Proportion of fixations on objects in VE compared to on terrain/sky | . . . | X | 16 | 12 |
| **Saccade Rate** | Total number of saccades per second | . . . | X | 16 | 12 |
| **Peak Saccade Velocity** | Average angular speed of eye movement during the saccade | . . . | X | 16 | 12 |
| **Saccade Magnitude** | Average angular distance of the saccade in degrees | . . . | X | 16 | 12 |
| **Blink Rate** | Summation of all blinks during the Math Task (or outside of the Math Task) divided by the total time of that time period | . . . | X | 16 | 11 |
| **Position Velocity** | Average change in position of avatar in the VE (in approximated meters) per second | . . . | X | 16 | 12 |
| **CNN Output** | Average P300-*like* response 1) comparing fixation on targets and distractors, 2) response for fixations, and 3) between groups and objects (targets, distractors) | X | . . . | 21 | 15 |
| **Change in CNN Output** | Average CNN Output for refixations subtracted from average CNN Output for initial fixations on each object | X | . . . | 21 | 13 |
