# Supplementary material for: Evidence of elevated situational awareness for active duty soldiers during navigation of a virtual environment
Source: PLoS One. 2024 May 10;19(5):e0298867. doi: 10.1371/journal.pone.0298867 (PMC11086823; doi:10.1371/journal.pone.0298867)
Supplement: S2 Table — The detailed F-statistics for the separate ANOVAs used to determine the effect of Military Status and Math Task and their associated degrees of freedom and p-values. (DOCX) [file pone.0298867.s002.docx]

| **Variable** | **Two-Way ANOVA** | | | | | |
| --- | --- | --- | --- | --- | --- | --- |
|  | **Military Status** | | **Math Task** | | **Military Status X**  **Math Task** | |
|  | **Wilks Lambda F, *df*** | ***p* -value** | **Wilks Lambda F, *df*** | ***p*-value** | **Wilks Lambda F, *df*** | ***p*-value** |
| **Duration of Individual Fixations (seconds)** | F (1, 25) = 0.00 | 0.957 | F (1, 25) = 1.25 | 0.274 | F (1, 25) = 3.42 | 0.076 |
| **Fixation Rate**  **(fixations per second)** | F (1, 26) = 0.03 | 0.866 | F (1, 26) = 0.44 | 0.512 | F (1, 26) = 4.86 | **0.037*** |
| **Object Rate**  **(objects per second)** | F (1, 26) = 0.31 | 0.581 | F (1, 26) = 16.92 | **0.000**** | F (1, 26) = 1.65 | 0.210 |
| **Proportion of Fixations on Object in the VE** | F (1, 26) = 2.57 | 0.121 | F (1, 26) = 0.58 | 0.452 | F (1, 26) = 3.02 | 0.094 |
| **Saccade Rate**  **(saccades per second)** | F (1, 26) = 3.18 | 0.086 | F (1, 26) = 8.62 | **0.007**** | F (1, 26) = 3.56 | 0.071 |
| **Saccade Magnitude**  **(degrees)** | F (1, 26) = 14.39 | **0.001**** | F (1, 26) = 4.03 | 0.055 | F (1, 26) = 1.09 | 0.307 |
| **Peak Saccade Velocity**  **(degrees per second)** | F (1, 26) = 17.29 | **0.000**** | F (1, 26) = 14.13 | **0.001**** | F (1, 26) = 0.00 | 0.991 |
| **Blink Rate**  **(blinks per second)** | F (1, 25) = 3.49 | 0.073 | F (1, 25) = 18.83 | **0.000**** | F (1, 25) = 0.48 | 0.496 |
| **Position Velocity (meters per second)** | F (1, 26) = 0.04 | 0.842 | F (1, 26) = 20.20 | **0.000**** | F (1, 26) = 0.23 | 0.638 |

**p < 0.05, **p < 0.01*
